# The Impact of Kinesiology Taping on a Greek Foot with a Hammertoe—A Case Report

**DOI:** 10.3390/healthcare9091178

**Published:** 2021-09-07

**Authors:** Tobiasz Żłobiński, Anna Stolecka-Warzecha, Magdalena Hartman-Petrycka, Barbara Błońska-Fajfrowska

**Affiliations:** Department of Basic Biomedical Science, Faculty of Pharmaceutical Sciences in Sosnowiec, Medical University of Silesia in Katowice, 41-205 Sosnowiec, Poland; astolecka@sum.edu.pl (A.S.-W.); mhartman@sum.edu.pl (M.H.-P.); bbf@sum.edu.pl (B.B.-F.)

**Keywords:** lesser toe deformity, hammer toe, Kinesiology Taping, foot, anthropometry of foot

## Abstract

Background and Objectives: Hammertoe, one of the most common toe deformities, causes pain due to overloading of the periarticular tissues and skin lesions. Additionally, it results in problems with footwear choice, an unattractive foot appearance and a deterioration in quality of life. The most common treatment for rigid and advanced deformities is surgery, and these procedures are widely described in literature. If the changes in the interphalangeal and metatarsophalangeal joints are flexible (that mean they undergo correction without causing pain) or surgery is not possible, conservative treatment should be considered. No research, however, has been found detailing this treatment method. Conservative treatment includes the Kinesiology Taping (KT) method, which involves applying taping to correct deformities. This report describes the effect of KT treatment in a female patient with hammertoes. Materials and Methods: Anthropometric foot measurements (3D scanner) and foot loadings (baropodometric platform) are presented before KT applying, immediately after tapes application and after tapes removal following one month of use. Results: After using KT application parameters such as: foot length, maximum foot load, load under the area of the metatarsals II-III changed. Conclusion: Kinesiology Taping seems to be a symptomatic form of treatment of the effects of lesser toes deformity, therefore it is a good alternative for patients who do not want or cannot undergo surgery.

## 1. Introduction

Hammertoe is characterised by a bend in the proximal interphalangeal (PIP) joint, with compensatory hyperextension (dorsiflexion) in the metatarsophalangeal (MTP) joint and distal interphalangeal (DIP) joint [1]. Initially, toe deformities are flexible (full correction is possible), however, as the deformity worsens, contracture of the periarticular structures may occur, resulting in the formation of rigid and non-correctable deformities [1].

Research involved 2662 Turkish adults estimating the incidence of smaller toe deformities at 8.9% [2]. Studies in the USA (2445 adults) estimate the frequency of hammertoe at 18% and claw toe at 2% [3]. Incidence increases with age (geriatric patients up to 50%) and deformities are five times more likely in women [4,5]. Other predisposing factors include poorly chosen footwear (too short, too loose and high-heeled shoes), increased length of the second toe and metatarsal II, family predisposition, hallux valgus, instability of or arthritis in the MTP joint, rheumatoid arthritis, amputation of the hallux and neurological diseases producing spasticity in the toes [1,2,4]. Most authors agree, however, that the problem is multifactorial [2,4,6].

The most common symptoms of deformities in the smaller toes include pain, mainly in the area of the MTP joints, including plantar plate injury, which is associated with overloading of the periarticular tissue, and skin lesions, e.g., corns and nail problems, as well as difficulties in finding appropriate footwear and an unsightly foot appearance causing embarrassment whenever the feet are exposed [1,3,5]. López-López in his research indicates the impact of the foot problem, including hammer toe deformation, on the quality of life [7,8]. His study confirms also that older adults with lesser toe deformity have lower scores in quality of life related to foot health, regardless of gender. Therefore, proper care and control of foot health may be extremely important in order to prevent the appearance or development of lower toe deformity [7,8]. Surgical treatment includes many different techniques such as PIP Joint Arthoplasty, Arthrodesis of the Proximal Interphalangeal Joint, Flexor to Extensor Tendon Transfer, Metatarsal Shortening (Weil) Osteotomy [1].

The Kinesiology Taping (KT) method is a therapeutic method using flexible tapes applied to selected parts of the body to relieve pain and improve circulation as well as to correct the position of different segments of the body [9]. The advantage of the tape is its continual effect 24/7 and the fact that the application remains on the skin for several days [10]. Each tape application should be preceded by an examination of the patient and an assessment of the musculoskeletal system [10]. The KT technique can be helpful in hip [11], knee [12], foot instability [13] or HV deformity pain syndromes [14]. KT might be used as an independent method of treatment or as the one element of therapy [10].

In the case of deformation of hammer toes KT supports the function of the weakened flexor digitorum brevis muscle and causes shortening of the stretched joint capsule [14]. The application mechanically forces the correct position of the toe in a standing position, while walking and in rest position [14].

The aim of the case study was to evaluate and compare the impact of the Kinesiology Taping application on the possibility of correcting hammer toe deformity as a document of the possibility of conservative treatment. While this is documented in one case, it may be of particular importance to clinicians such as orthopedic surgeons, physical therapists, podiatrists, and other foot professionals. This aim is part of the search for effective conservative treatment that will help patients who are not qualified for surgery for various reasons.

So far, there is no research documenting the effect of Kinesiology Taping on hammer toe deformation. The purpose of this study is to assess the changes in anthropometric parameters using a 3D scanner in order to document the effect on the position of the flexed toe, as well as to assess the impact of patch application on the way the individual parts of the feet are loaded while standing and walking.

## 2. Materials and Methods

A woman, aged 75, BMI = 21 kg/m^2^ had a bilateral hammertoe deformity of the second and third toes. The patient reported pain in the area of the toes and forefoot lasting for several years, periodically increasing. The patient reported daily pain of 4 points on the Visual Analogue Scale, accompanying the deformity of the second toe of the right foot (RF) mainly when walking in footwear. There was redness and abrasions on the dorsal aspect of the toes level with the PIP joints. The second toe was longer in both feet (Greek foot). A manual examination revealed full mobility of the MTP and DIP joints and slight stiffness in the PIP joints in dorsiflexion, however there was a full range of motion. A slight tenderness was noticed when the area around the plantar plate was palpated. The Dorsal Drawer Test, The Plantar Plate Provocation Test and The Digital Plantarflexion Test [5] did not cause any pain (a negative result). During the assessment of the musculoskeletal system no disturbances in the biomechanics of the lower limb axis were found.

In the case of the patient deformities were elastic enough (range of motion in the interphalangeal and metatarsophalangeal joints allows you to correct the position of the toe without causing pain) to try conservative treatment using manual therapy techniques and the Kinesiology Taping method.

**Figure 1:**
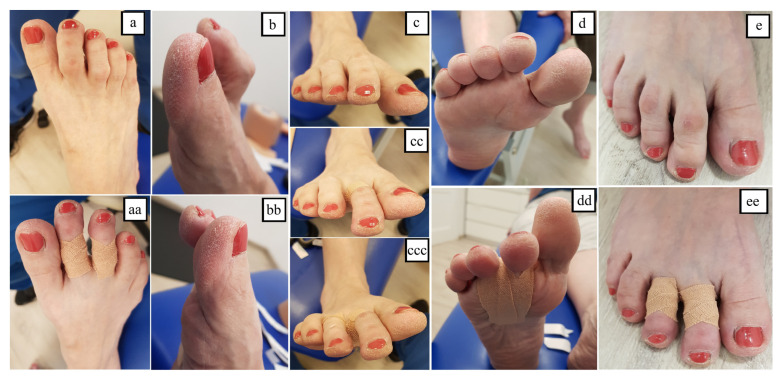
Hammertoes on right foot: dorsal (**a**), lateral (**b**) anterior (**c**), plantar (**d**) and standing (**e**) views. Hammertoes after KT application: dorsal (**aa**), lateral (**bb**), anterior—after application on second (**cc**) and third toe (**ccc**), plantar (**dd**) and standing (**ee**) views.

The patient was examined before applying the taping (T0), immediately after its application (T1) and after 32 days of using Kinesiology Taping (T2). The patient was asked to remove the tape application in the evening before examination day. Tests were conducted on the FreeMED MAXI (Sensor Medica, Guidonia Montecelio, Italy) baropodometric platform assessing how individual parts of the feet were loaded when standing (the examination included a 30-s standing analysis) and walking (the study included at least 10 steps for each foot). A 3D podoscanner (Sensor Medica, Guidonia Montecelio, Italy) was used for anthropometric measurements of the foot. Kinesiology Taping (KT) to correct the toes: the 2.5 cm wide 3NS TEX tape in a Y-shape was applied with the two tails around the proximal phalange without stretching, and the base on the plantar side of the foot with maximum stretch (corrective application 75–100%) (Figure 1). The taping was changed by physiotherapist every 3–4 days while patient’s therapy in the clinic after checking skin condition. Furthermore, during the same therapy dorsiflexion of the PIP joint was performed (traction and mobilization techniques) to improve the flexibility of the toes.

The research project was consistent with the Declaration of Helsinki and was approved by the Bioethical Committee of Medical University of Silesia (approval number: KNW/0022/KB1/27/I/16, Ethics Committee approval date: 6 June 2016). The patient signed a consent form to participate in the experiment.

## 3. Results

The length of the RF, measured at T1, increased by 3 mm and the left foot (LF) by 4 mm, judged to be the effect of the mechanical straightening of the toe. The T2 measurement showed a 5 mm increase in the RF length and 4mm in the LF compared to the T0 test. In the static test the T2 measurement showed that the maximum foot load was reduced by 50 g/cm^2^ (LF) and 62 g/cm^2^ (RF), and the forefoot load decreased by 8% (LF) and 9% (RF), but the average load per foot had not changed significantly. In the dynamic test conducted at T2, the maximum load was reduced by 236 g/cm^2^ (LF) and 164 g/cm^2^ (RF) and the average load by 70 g/cm^2^ and 67 g/cm^2^, respectively. The support surface of both feet increased by 6 cm^2^. In the gait dynamic test on a baropodometric platform the T2 measurement, with the foot divided into 9 areas, showed an increase in the area of the smaller toes of 3.25 cm^2^ (LF) and of 4 cm^2^ (RF), and of the central part of the forefoot corresponding to metatarsals II and III by 3.5 cm^2^ and 2.25 cm^2^, respectively. At T1 the load on the smaller toes decreased by 3.6% (LF and RF), while the load under the area of the metatarsals II-III decreased by 2.32% and 3.66%. The patient’s pain symptoms decreased from 4 to 1 on the Visual Analogue Scale and no longer occurred daily despite no change to the footwear (during the therapy, the patient wore the same shoes that she used before starting the therapy). Table 1 and Table 2 show the results.

## 4. Discussion

The purpose of this case report is to present this novel therapeutic approach which demonstrates the effectiveness of using KT to change the position of the toe and foot and to change the load on parts of the plantar of the foot in both static and dynamic conditions in a person suffering from hammertoes. A hammertoe deformation can be caused by many factors, including incorrectly selected, too tight shoes, genetic predisposition, excessive body weight, coexistence of hallux valgus causing pressure on the second toe [2,3,15,16]. One of the predisposing factors for the formation of the hammertoe in the second toe is the increased length of metatarsal II, typical of so-called Greek foot [17]. Fleischer [17] reported that a longer metatarsal II is the cause of increased load in the MTP joint and metatarsal head, which may predispose to plantar plate injuries preventing excessive movement between the bones, particularly hyperextension of the MTP joints. Weber [18] confirm the association of a longer metatarsal II with increased pressure under its surface. Hulstaert [19] point to the relationship between a deformity in the smaller toes and damage to the plantar plate, usually accompanied by damage to the collateral ligaments. Initially, this produces metatarsalgia and oedema, which develops into incorrect toe positioning—a hammer, mallet, claw and crossover toe, and a disruption to the biomechanics of the toe. In this study, the Greek foot showed a greater load under the metatarsals II-III, which concurs with the studies of the abovementioned authors, and may have contributed to the deformity. The use of KT correcting the hammertoe position reduced the load on this area, preventing the occurrence of metatarsalgia, overloading of soft tissues and skin lesions in the form of calluses and corns. Despite the decrease in load under the metatarsals II–III, both at T1 and T2, the load remained higher than under metatarsals I and IV–V, which may be associated with the anatomical length of the bone itself.

A patient with a Greek foot was selected for the analysis of the KT effectiveness, because it allowed an evaluation of the change in position of the second toe by measuring the length of the foot. Podoscanner 3D estimates the length of the foot on the basis of its two endmost points, so when the toe is longer, a difference in the length of the foot can be determined, because the distance between the shorter second bent hammertoe and the heel will not be taken into account [20].

Many studies show the positive effect of KT on the musculoskeletal system [11,13,21,22] indicate an improvement in the functioning of the joints of the lower limb and the resulting improvement in the stability of the ankle. Various authors show that Kinesiology Taping can be used to combat pain symptoms associated with musculoskeletal injuries [11,21]. Kinesiology Taping supports injury prevention [22] and improves lymphatic flow [9].

There is no data confirming the effectiveness of conservative treatment with using KT in case of deformity in the smaller toes. Kinesiology Taping is a symptomatic form of treatment of the effects of lesser toes deformity, therefore it is a good alternative for patients who do not want or cannot undergo surgery. The limitations of our study include one case, in the study we focused on the assessment of changes in the anthropometric parameters of the foot, parameters of the static and dynamic load of the foot. However, there is the need for additional research on a group of patients, followed long-term, to evaluate the efficiency of using Kinesiology Taping method in treatment patients with hammer toe deformities. While this is documented in one case, it may be of particular importance to clinicians such as orthopedic surgeons, physical therapists, podiatrists and other foot professionals. A case-control study and a sample of people from other countries would improve the strength of this research. The patient’s reaction to conservative treatment suggests that in this case the regular use of manual therapy and the Kinesiology Taping method will maintain the effects of the therapy in terms of pain reduction.

## 5. Conclusions

Kinesiology Taping is a technique that seems to have the short-term effect of changing the position of small toes in patients suffering from hammertoe. The application of the taping on the lesser toes proves to the patient that the correction in the position of the toes has a positive effect on the everyday functioning of the foot and on the reduction of pain while the tape is on the patient’s toe. Therefore, those patients, who for some reason, do not want or cannot undergo corrective surgery, can benefit from this conservative treatment option and thus improve the quality of their lives. However, there is the need for additional research on a group of patients, followed long-term to evaluate the efficiency of using Kinesiology Taping method in treatment patients with hammer toe deformities.

## Figures and Tables

**Table 1:** Selected anthropometric, static and dynamic foot parameters taken before using the Kinesiology Taping (T0), directly after application of the tapes (T1) and after tapes removal following monthly using (T2).

Types of Analyses	Parameter	Left Foot			Right Foot		
		T0	T1	T2	T0	T1	T2
3D scan analyses	Foot length [mm]	262	266	266	265	268	270
	Foot width [mm]	90	90	90	93	93	93
	Heel width [mm]	68	68	68	68	68	68
	Length of the longitudinal arch of the foot [mm]	192	192	192	195	195	195
	Metatarsal head I–Vwidth [mm]	65	65	65	66	66	66
	Heel angle [°]	1	1	1	1	1	1
	Knee angle [°]	3	3	3	3	3	3
Static analyses	Surface of the foot [cm^2^]	161	166	164	156	159	156
	Surface of the forefoot [cm^2^]	85	92	88	86	89	90
	Surface of the hindfoot [cm^2^]	76	74	76	70	70	69
	Max. foot load [g/cm^2^]	486	437	436	505	423	443
	Mean load [g/cm^2^]	216	222	234	257	250	255
	Forefoot load [%]	52	44	44	54	43	45
	Hindfootload [%]	48	56	56	46	57	55
Dynamic analyses	Max. load [g/cm^2^]	1440	1252	1204	1448	1308	1284
	Mean load [g/cm^2^]	435	323	365	462	408	395
	Surface of the foot [cm^2^]	196	199	202	212	216	218
	Length of foot [mm]	270	280	280	280	280	290
	Forefoot load [%]	63	60	60	64	60	59
	Hindfoot load [%]	37	40	40	36	40	41
	Load on medial part of the foot [%]	48	48	53	44	47	44
	Load on lateral part of the foot [%]	52	52	47	56	53	56

**Table 2:** Surface and load of foot areas before using the Kinesiology Taping (T0), directly after tapes application (T1) and after tapes removal following monthly using (T2).

	Left Foot						Right Foot					
	Surface [cm^2^]			Load [%]			Surface [cm^2^]			Load [%]		
Foot Area	T0	T1	T2	T0	T1	T2	T0	T1	T2	T0	T1	T2
Hallux	23.25	23.75	23.75	10.36	10.77	10.23	21.50	21.50	21.25	12.00	12.14	13.02
Toes II-V	18.50	22.00	21.75	13.41	8.27	9.81	20.00	23.75	24.00	9.95	5.78	6.34
Metatarsal I	13.50	13.25	13.75	8.20	8.13	8.70	16.00	16.00	15.75	7.93	8.60	8.33
Metatarsal II–III	19.00	21.75	22.50	14.59	11.21	12.27	26.00	28.00	28.25	14.79	11.55	11.13
Metatarsal IV–V	20.00	20.00	20.50	8.07	9.78	9.75	30.75	29.50	29.75	11.55	9.98	10.85
Medial arch	22.50	22.00	22.25	8.27	8.79	8.13	20.25	20.25	20.50	4.98	6.79	5.97
Lateral arch	25.00	24.25	24.50	16.18	17.35	17.26	25.25	25.75	25.75	10.99	13.98	12.74
Medial heel	24.50	24.25	24.25	11.10	12.55	12.25	23.25	23.00	23.25	12.88	15.58	15.55
Lateral heel	29.75	28.25	28.50	9.90	13.04	11.53	28.75	28.50	29.00	15.03	15.57	15.98

## Data Availability

The data presented in this study are available on request from the corresponding author. The data are not publicly available due to the presence of sensitive data in each survey form.
